# News from Societies

**Published:** 1955-09

**Authors:** 


### *News from Societies*

### TORQUAY AND DISTRICT MEDICAL SOCIETY

### "Collaboration between Church and Medicine"

As a result of a public pronouncement by the Archbishop of Canterbury on the participation of the clergy in the art of healing, it was determined that a meeting of interested members of the respective professions concerned should be held in the South Devon area, not only with a view to discussing the subject itself, but also with a view to taking practical steps both to identify the ground common to the Church and Medicine and to indicate whether co-operation could be effected. Such a meeting was called at the Torbay Hospital on February 26th, 1953, on the initiative of the Torquay and District Medical Society, on which occasion the local clergy joined the Society in the meeting, forty-five clergy and forty-one doctors being present. The Bishop of Exeter opened a discussion on the place of clergy and doctors in Medicine by pointing out that there was a great need for co-operation between clergy and doctors, for they were both serving in the same ministry. Human nature was a unity composed of body and soul, and because of this unity priests and doctors must work together; their spheres might be distinct and each might have his own expert knowledge, but their objectives were closely interrelated. There was a great temptation for both to arrogate to themselves healing power that belonged to God, whereas at best, in both the spiritual and physical sphere, they were merely channels of grace. The first requirement was a profound humility, realizing that their duty was to create conditions favourable for operation of the Divine power. The Bishop then proposed three topics for discussion. In regard to the views he expressed, he said he was speaking for himself and not for the Church. Of euthanasia he said it was wrong to take life; but it was a doctor's duty to relieve unnecessary pain and it might well be that the drug needed to achieve this might shorten life. Of abortion he said that the doctrine of the Roman Church was that from the moment of conception the embryo had equal rights with the mother. He himself thought that the problem hinged on whether we regarded the unborn baby as having a distinct personality. The infant in the womb certainly had a claim to life; but had it a greater claim than its mother? He thought that if there was a good reason for believing that the continuance of pregnancy would be fatal for the mother, to kill the foetus might be the lesser of two evils and therefore justifiable, especially when to do nothing would mean the death of both. On telling the patient that his end was near, the Bishop thought that it was unfortunate that in recent times the clergy had ceased to consider it their duty to include in their ordinary course of religious instruction the preparation for death. It was so much easier to tell the truth to patients so prepared, and so the best use could be made of their last few days of life. It was of the utmost importance that chaplains should be kept informed of those on the danger-list and be allowed free access to those who were likely to appreciate their administrations.

Many clergy and doctors took part in the discussion which continued for about two hours. A strong feeling became evident that some means should be provided for maintaining a liaison between clergy and doctors in the area. The Bishop welcomed this idea and closed the meeting by charging the Rural Dean to collaborate with the Council of the Medical Society in selecting a small committee of clergy and doctors to consider how this purpose could best be achieved.

Such a committee was constituted later and discussions have taken place on subjects of common interest. It was soon evident that the most fruitful field would be that pertaining to the emotional state of the patients' minds and relating to their thought processes. There was a meeting held in April at which the problem of pain was discussed; and at a subsequent meeting the problem of fear was debated. There were two openers of this discussion: Dr. Guy Tanner (Newton Abbot) and the Rev. Mr. T. P. Vokes-Dudgeon.

Dr. Tanner said: "A famous physician used to say that it was always fear that drove a patient to see his doctor—fear for his future health, fear for his ability to earn his living, perhaps even fear for his life—and that the doctor who failed to recognize and treat this fear was only doing half his job.

"The patient comes to us doctors with a set of symptoms. They may indicate trouble which is neither dangerous nor disabling. On the other hand, they may be the first signs of a downhill journey with little hope of recovery. They may foretell pain, loss of earning power, the prospect of surgical operations. They may be themselves the result of fear as well as its cause. So many people come to us with physical symptoms due solely to mental anxiety, whose real trouble is a failing business or an impending family catastrophe. During the War we met these same symptoms caused by the fear of danger which was only too obvious.

"These, then, are the sort of fears which people have to face. The question is, what can we do to help?

"The best thing we can do, and fortunately it is possible in the great majority of cases, is to remove the fear altogether. We do this by reassuring the patient that his malady is not serious and will soon pass. If we cannot do this, if we know that serious trouble is indeed present, we are faced with a difficult problem and a heavy responsibility and that is to decide how much to tell. Under what circumstances and to what extent, are we justified in concealing from a sick person the danger of his or her condition? I will leave the meeting to discuss this problem amongst others.

"We shall, then, be able to remove fear in many cases. We shall feel justified in some others in reducing fear by hiding or understating the danger. There remain many more in which the danger is only too obvious, and fear is inevitably present. Our task must then be to help the affected person to face his fear, to live with it without being overcome by it, and if possible to conquer it.

"We can help in three ways, and the first is by the use of drugs. We can soothe by various members of the barbiturate family and in extreme cases by morphia, and we can boost morale by benzedrine and its derivatives. The patient himself may use alcohol. I will only say this of drugs in the treatment of fear; they are often useful and sometimes essential in an emergency, to help a patient over a bad patch of trouble; but as a long-term policy they are a confession of failure. Fear is a malady of the mind and spirit, and it is through the mind and the spirit that lasting help must come.

"The second way in which we can help is by tackling the patient's mind. Every general practitioner has to be a practical psychologist of sorts, and we do try to talk our patients into a more calm, patient and optimistic frame of mind in the face of their difficulties and fears. But many states of fear, particularly those which are morbidly exaggerated, do sometimes benefit by treatment by a proper psychiatrist; and in one type of fear, the delusional type, this is quite essential. I mean by this the sort of person who is convinced that his neighbour is dropping bombs through his letter-box or poisoning his drinking-water. His fear is madness and only a psychiatrist can hope to tackle him adequately.

"The third way, and to my mind by far the most important way and the best, is through the patient's spirit. Here the family doctor, if he is willing, can help a great deal, the parish priest or minister much more, and co-operation between doctor and priest most of all. If the patient is known to be a religious man, the approach should be easy. If the doctor is himself a convinced and practising Christian, and especially if he has learnt to talk about his own religious faith naturally and without embarrassment, then again the approach should be easy. If the doctor is shy about religion or the patient antagonistic (and this latter state is extremely rare in my experience) a direct approach may be difficult; but at least the patient's relations should be reminded of the help that a parish priest or minister is ready and anxious to give. The important thing for us doctors to remember is the fact, proved over and over again by history and human experience, that the Christian faith can enable a human being to face the greatest dangers in peace of mind and relief from fear. Many of us have the great good fortune to know this from personal experience in our own lives or in the lives of those with whom we have had contact. To the sceptics, if there are any present, I would quote a statement by Sir Heneage Ogilvie in a recent article in *The Practitioner*. He says: " The most effective weapon in the treatment of the troubled spirit may be the faith in a power higher than ourselves." This " may be " sounds like the statement of a sceptic who is becoming sceptical of his own scepticism! Anyway, whether we say " may be " with Ogilvie or " is " with conviction, we should at least offer this greatest of all comforts to those who come to us for help. We can do so by recognizing our brothers of the clergy as specialists in faith, and calling in their assistance much more often than we do at present.

The Rev. Mr. T. P. Vokes-Dudgeon, (Vicar of St. Marychurch, Torquay) spoke of fear, and of its evil effects. Religion replaced fear by hope, and even death became less frightening. Mr. Vokes-Dudgeon explained his views on the place of Baptism and the Eucharist in easing man's fear, and how some people might be relieved by confession and absolution. He said that the Sacrament of Unction brought calm to very sick people. No two patients were precisely alike and the priest must use patience and discrimination. Moreover, he should not dabble in the antics of the psychiatrist.

